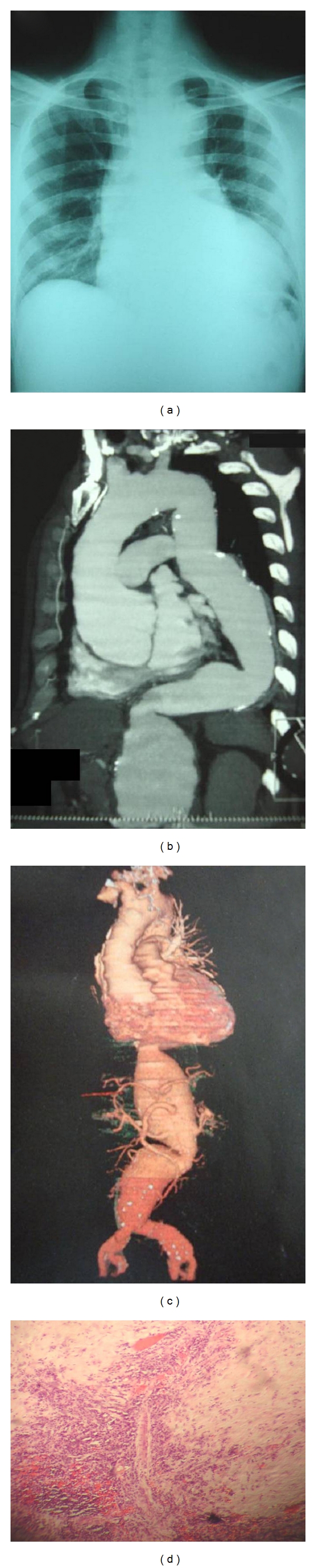# Syphilitic Aortic Aneurysm in a Young HIV-Infected Man: Case Presentation

**DOI:** 10.1155/2011/935271

**Published:** 2011-09-15

**Authors:** Juan Carlos Cataño, Isabel Cristina Ramirez

**Affiliations:** Infectious Diseases Section, Internal Medicine Department, University of Antioquia Medical School, Carrera 65D # 34 A—35, Medellín, Colombia

## Abstract

We describe the case of a young HIV-positive man who presented to the emergency room in hypovolemic shock. During subsequent evaluation, we documented a huge aortic aneurysm consistent with tertiary syphilis. The final autopsy demonstrated the extent of cardiovascular compromise caused by this aneurysm.

## 1. Case Presentation

A 35-year-old male patient with a 10-year history of HIV presented to the emergency room complaining of 3 months of progressive dyspnea and 2 hours of sudden chest pain and profuse diaphoresis. His most recent CD4 count on record was 154 cells/mL with a HIV viral load of less than 40 copies/mL. He had been taking zidovudine-lamivudine and efavirenz for the past 8 years. On physical examination, he was afebrile, anicteric, and without rash. His arterial pressure was 70/40 with a heart rate of 120 beats per minute. There was no prominent lymphadenopathy. The lungs were clear, but on cardiac auscultation a grade IV systolic murmur was auscultated at the left sternal border. On abdominal examination, a pulsatile epigastric mass was palpated. Cardiac enzymes were not elevated. The EKG was consistent with left ventricular hypertrophy without signs of cardiac ischemia. A chest radiograph demonstrated mediastinal widening (Figure 1(a)) and prompted a 3D computed tomography of the aorta (Figures 1(b) and 1(c)), which revealed an aortic aneurism extending from the ascending aorta to the iliac vessels. Further laboratory studies were notable for a strongly positive Venereal Disease Research Laboratory (VDRL) titer of 1 : 128 and a positive fluorescent treponemal antibody absorption (FTA-ABS) test. The patient denied any knowledge of prior syphilis infection or testing. The patient's aortic aneurysm was presumed to be secondary to syphilis. Waiting for cardiovascular surgery consultation, during the following 24 hours, the patient was started on aqueous crystalline penicillin and volume resuscitated with a crystalloid infusion. During the second hospital day, the patient developed sudden abdominal pain and hypovolemic shock. He died 30 minutes later despite resuscitation. An autopsy was performed on the following day, and 3500 cc of blood was encountered within the peritoneum as well as a ruptured abdominal aneurysm. On histopathological examination of the aorta, the adventitia showed focal cellular infiltrates with a predominance of lymphocytes and the vasa vasorum demonstrated thickening of the intima (Figure 1(d)) strongly suggesting the diagnosis of a syphilitic aortic aneurysm. 

## 2. Discussion

Since the 1980s, the world has been facing a HIV epidemic and we have been learning about the different presentations of opportunistic infections associated with AIDS; one of the most common sexually transmitted diseases (STDs) associated with HIV is Syphilis, in which clinical manifestations can vary widely. 

Syphilis is a bacterial STD caused by the spirochete *Treponema pallidum*, subspecies *pallidum*, which had developed as a new world disease out of evolving treponemal species [1]. The disease progresses through primary, secondary, and tertiary phases. Involvement of the cardiovascular system is the most dangerous sequela of the tertiary phase (late syphilis). This usually manifests as syphilitic aortitis and infrequently as gummatous myocarditis. The advent of effective antibiotic therapy affected a pronounced decline in the incidence of late cardiovascular syphilis in developed countries where syphilitic aortitis has been relegated to the category of rare cardiovascular disease [2]. In developing countries, like Colombia, syphilitic aortitis remains a frequent cause of nonatherosclerotic aortic disease found at autopsy.

In untreated syphilis, aortitis may manifest 10 to 40 years after the initial sexual contact [2]. The ascending aorta is most often affected (in 50% of cases), followed by (in decreasing order of incidence) the aortic arch, the descending aorta, and the abdominal aorta. Impairment of coronary ostia and aortic valve is also possible [3, 4]. The main cause of death in approximately 80% of cases is rupture of saccular aneurysms, which requires surgical intervention [4].

In the natural course of cardiovascular syphilis, the primary infection is followed by *T. pallidum* invasion of the aortic wall, initially within the adventitia and soon thereafter in the lymphatic vessels. The rich lymphatic system of the ascending aorta is one of the main reasons for the tropism of spirochetes there [5]. The vasa vasorum then undergoes a process of endarteritis obliterans, necrosis of medial layer (mesarteritis), and infiltration of plasma cells. Consequently, the elastic tissue of the vessel is destroyed and replaced by scar tissue. The inflammatory process may continue for as long as 25 years after the initial infection [6]. The initial clinical presentation may then be of angina when there is obstruction of the coronary ostia or dyspnea when there is aortic valve incompetence or compression of the respiratory organs. However, the most common clinical symptom is chest pain secondary to rapid expansion of the luetic aneurysm [2–5, 7]. This case describes an abnormally advanced syphilitic aortic aneurysm for such a young patient, a condition likely exacerbated by his HIV infection [8]. The rupture of this aneurysm resulted in hypovolemic shock and sudden death, a tragedy, which could have been prevented with existing treatment, adequate screening, and additional resources.

##  Conflict of Interests

The authors declared that there is no conflict of interests.

##  Consent

The patient's mother signed the informed consent for the case report to be published.

## Figures and Tables

**Figure 1:**